# Images as catalysts for meaning-making in medical pain encounters: a multidisciplinary analysis

**DOI:** 10.1136/medhum-2017-011415

**Published:** 2018-06-12

**Authors:** Deborah Padfield, Helen Omand, Elena Semino, Amanda C de C Williams, Joanna M Zakrzewska

**Affiliations:** 1 Slade School of Fine Art, University College London, London, UK; 2 Institute of Medical and Biomedical Education, St George’s, University of London, London, UK; 3 Department of Social, Therapeutic and Community Studies (STACS), Goldsmiths University of London, London, UK; 4 Department of Linguistics and English Language, Lancaster University, Lancaster, UK; 5 Research Department of Clinical, Educational and Health Psychology, University College London, London, UK; 6 Pain Management Centre, National Hospital for Neurology and Neurosurgery, Eastman Dental Hospital, London, UK

**Keywords:** pain management, art and medicine, fine art, arts therapist, metaphor

## Abstract

The challenge for those treating or witnessing pain is to find a way of crossing the chasm of meaning between them and the person living with pain. This paper proposes that images can strengthen agency in the person with pain, particularly but not only in the clinical setting, and can create a shared space within which to negotiate meaning. It draws on multidisciplinary analyses of unique material resulting from two fine art/medical collaborations in London, UK, in which the invisible experience of pain was made visible in the form of co-created photographic images, which were then made available to other patients as a resource to use in specialist consultations. In parallel with the pain encounters it describes, the paper weaves together the insights of specialists from a range of disciplines whose methodologies and priorities sometimes conflict and sometimes intersect to make sense of each other’s findings. A short section of video footage where images were used in a pain consultation is examined in fine detail from the perspective of each discipline. The analysis shows how the images function as ‘transactional objects’ and how their use coincides with an increase in the amount of talk and emotional disclosure on the part of the patient and greater non-verbal affiliative behaviour on the part of the doctor. These findings are interpreted from the different disciplinary perspectives, to build a complex picture of the multifaceted, contradictory and paradoxical nature of pain experience, the drive to communicate it and the potential role of visual images in clinical settings.

‘Pain exposes for all of us deep problems of meaning’ (Charon 2016).

## Background

The challenge for those treating or witnessing pain is to find a way of crossing the chasm of meaning between them and the person living with pain, navigating between the most certain thing in the life of one and paradoxically the very thing arousing doubt in others,^1^ exacerbated by the limitations of language and the unequal hierarchies of knowledge and agency in many contexts.

This paper proposes that images can strengthen agency in the person with pain, particularly but not only in the clinical setting, and can create a shared space within which to negotiate meaning. It draws on multidisciplinary analyses of unique material resulting from two fine art/medical collaborations in London, UK, in which the invisible experience of pain was visualised. In parallel with the pain encounters it describes, the paper seeks to weave together the insights of specialists from a range of disciplines whose methodologies and priorities sometimes conflict and sometimes intersect to make sense of each other’s findings. When integrated, these differing perspectives have the potential to reshape our understanding of what it means to live with and witness pain, and the capacity of images to enhance communication around pain.

To summarise the projects that generated the data,^2–5^ a series of photographs of pain were co-created by an artist with chronic pain patients in one-to-one workshops, in order to give visible and tangible form to subjective experience. The set of photographic images (pain cards) was used to explore whether they could be useful to other patients in future healthcare consultations. Both projects, *perceptions of pain*^6 7^ and *Face2Face*^5 8^ hypothesised that images placed between doctor and patient could improve communication and interaction. Ten pain clinicians volunteered to pilot the images in their consultations, two each using images and two without, the final numbers were 17 patients in the with image group and 21 in the without image (control) group. Patients (who had not been involved in making the images) were offered the pain cards in the waiting room, asked to select any that resonated for them and take them into their consultation to use if and how they liked.

Subsequently, a multidisciplinary team analysed the postconsultation questionnaires, video-recorded footage and transcripts.^2 9 10^ Through diverse methodologies, they found that images encouraged discussion of the emotional components of pain, and impacted on both verbal and non-verbal aspects of the interaction: images made the language more personal and increased affiliation behaviours, engaging both patient and doctor in more negotiated and democratised behaviour.^2 9 10^

This paper builds on these analyses. We took a short section of video footage chosen as an exemplar of the kind of way in which images were used in the pain consultations. The extract was selected as it picks up on prevalent themes emerging from the narrative analyses such as change in identity and emotional distress. Using a short section of the encounter allows us to examine the interactions in fine detail from the perspective of each discipline, consistently referring back to the filmed footage and to the patient’s own words. These perspectives provide insights into the lived experience of chronic pain, and shed light on the emotional and social impact of chronic pain and its very real challenges to identity. The process of writing the paper parallels the challenges of understanding, tolerating and integrating multiple interpretations that may coexist in the consulting room. Presenting multiple meanings contrasts with the illusion of objectivity in much clinical description. The presence and use of the pain cards highlighted the subjectivity of interpretation, particularly within the highly charged communication of healthcare settings. The authors argue that the multidisciplinary analytic process can illuminate the challenge of negotiating between different perspectives, and propose that images can act as a vehicle for navigating between different meanings in the clinic.

The paper is distinct in two ways: we tackle the issue of introducing a visual language into clinical pain consultations by means of groundbreaking interdisciplinary projects and we show the value of interweaving the methodologies and perspectives of an interdisciplinary group of scholars on the same data: an artist, pain specialist, psychologist, linguist and art-psychotherapist. Through combining them we develop a rich and multilayered account. We have not attempted to create a single voice but chosen to highlight these multiple voices in order to make the process and its findings transparent.

## Extract

**Figure 1:**
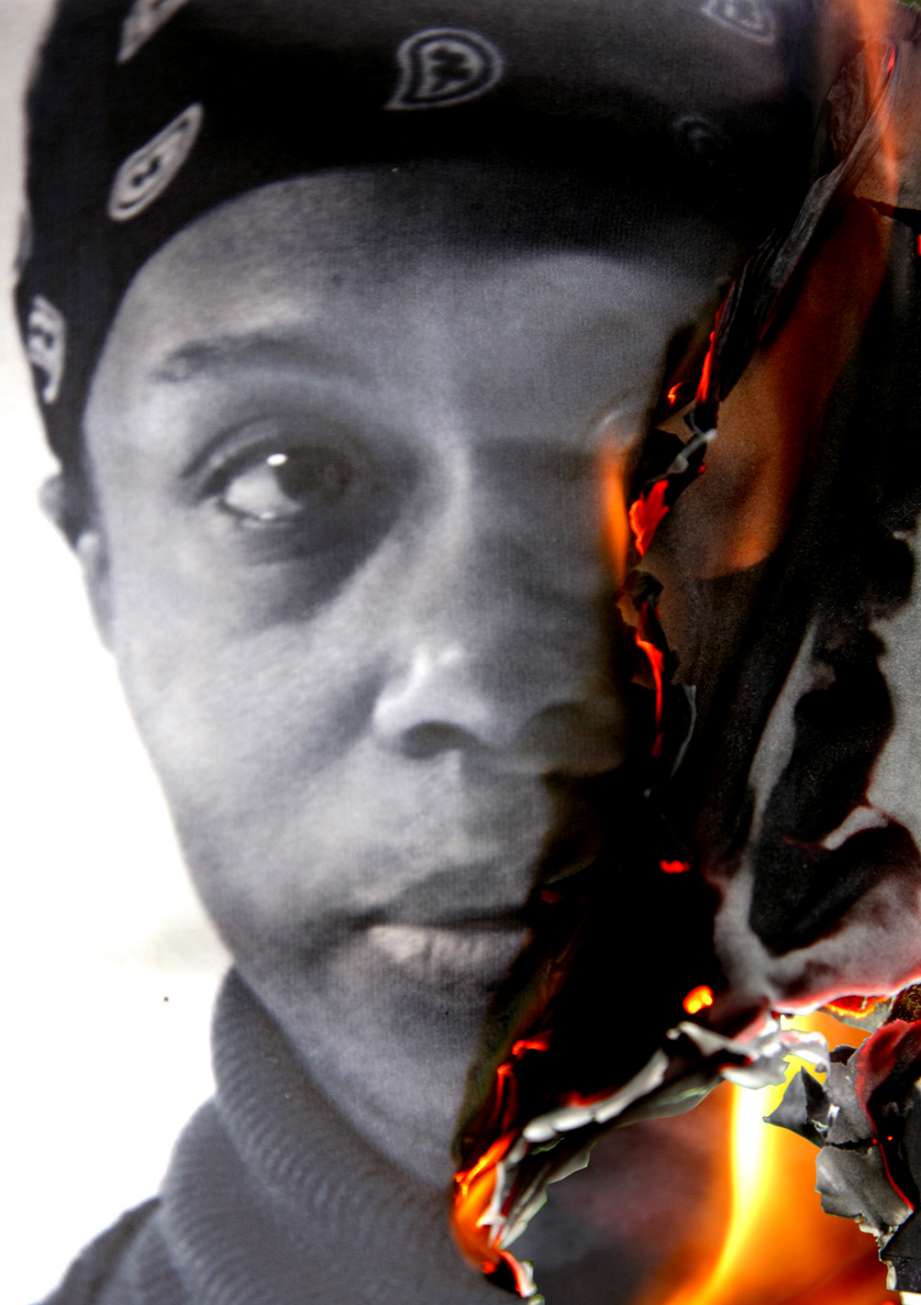
Image of pain co-created by Deborah Padfield with Linda Williams from the series Face2Face, 2008–2013.

1. PG5 […] Um, this has to do with my self-identity…
2. CG5 Ah, okay.
3. PG5 … being worn away by always having to pain manage and knowing that I have an achy time.
4. CG5 What about that one makes you think about your self-identity?
5. PG5 Because that person’s face is burning off.
6. CG5 Right, okay.
7. PG5 So for me that’s self-identity.
8. CG5 That’s interesting, actually, because, um, I did some work with, I think it was her [?], um, when I’m doing this project, so it helps me identify with that as well.
9. PG5 Yes, because for me, if that’s a portrait of a person, well you know…
10. CG5 Yes.
11. PG5 … I’m a visual, I work in visual…
12. CG5 Visual things as well.
13. PG5 So that’s like the burning off of… and plus my pain is hot (Figure 1).

## Analyses

The selected extract takes place towards the end of a consultation and involves an exchange between a female clinician and female patient. Both are Caucasian and aged between 41 and 59 years.

**Figure 2:**
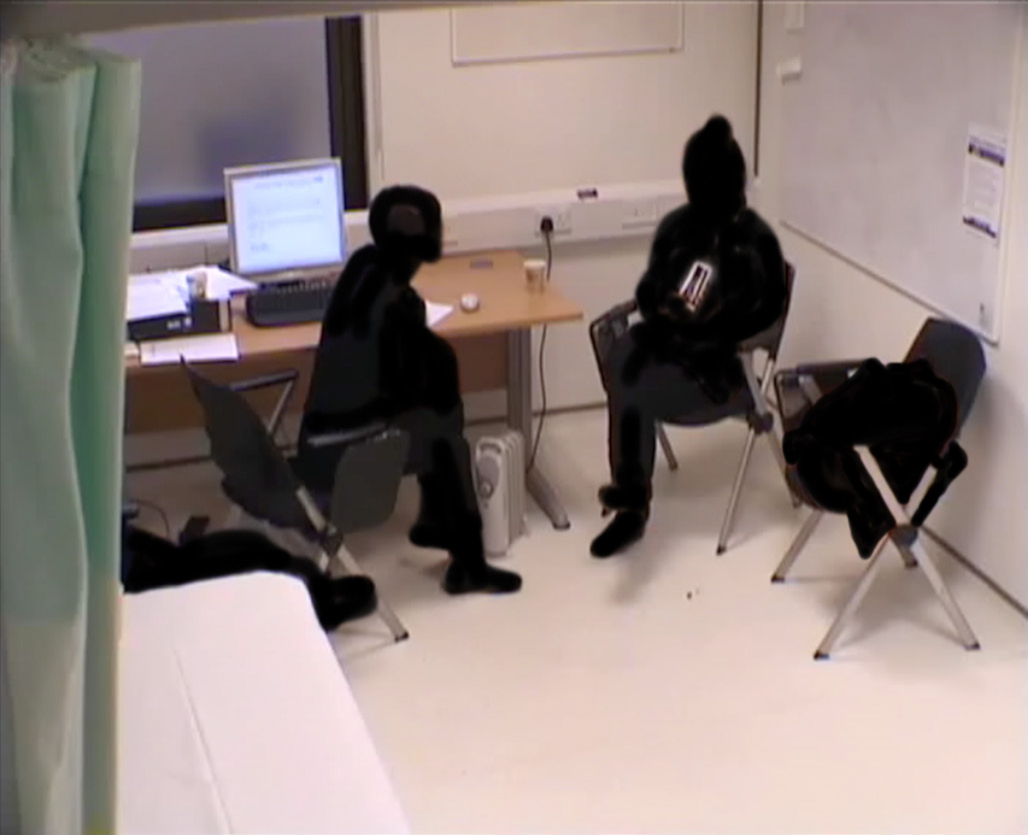
Anonymised screen grab of video footage from this consultation (filmed with consent) showing the non-verbal interaction.

Up until the beginning of the extract the patient has given details of her pain experience, much of which has been in some way identity-building. At the start of this extract, towards the end of the encounter, both patient and doctor are sitting with right leg crossed over left. The patient sits back in her chair but her head is very upright; she faces the clinician at about 120 degrees, with the corner of the desk only just intruding on the space between them. The doctor is on the edge of her chair (the same height and type as the patient’s), leaning forward facing the patient, a little bit hunched, head slightly to one side, left hand resting on desk and right on her knee. The computer is on and has been used earlier, but the doctor is sideways to it; her handwritten notes are more accessible than the keyboard but not in her gaze. The patient, a woman in her 40s, is describing how she uses illicit drugs to control her pain and at this sensitive moment picks up the cards (she has selected 10) and shuffles them, holding them close to her abdomen. However, she does not talk about them until invited by the doctor (for clarity we use the term ‘doctor’ to refer to the clinician in the consultation and ‘pain specialist’ to refer to the coauthor) (figure 2).

The coauthors all agree that the selected extract marks a shift in the participants’ relationship and a change in the dynamic between them. This is reinforced by the quantitative findings from both verbal and non-verbal analyses.^2 9 10^ However, they disagree on the emotional environment in which the extract falls, reading the emotional tone and meaning of the exchange slightly differently. This parallels the ways in which clinician and patient can experience a consultation in markedly different ways. The psychologist observed that the interview up until then had been ‘*quietly absorbed, with some questions from the clinician but the patient largely telling her pain story in her own way. Occasionally, the doctor has stopped her to use the computer, both looking at the screen. Nearly half an hour into the interview, the subject turns to possible causes of the pain, and the patient sits back while the doctor leans forward, taking more equal turns at talking. Then the patient describes “getting high” on the large amount of opioids that she takes, but assuring the doctor (when asked) that her GP is aware of it. During this very sensitive discussion, the doctor (already of a slighter build than the patient) hunches slightly, as if to make herself smaller and less threatening; the patient goes on to express disgust at the drug’s effect on her.*’

The artist noted that, at this same moment, ‘*the patient picks up the cards, that is, during this very sensitive and uncomfortable part of the consultation where she is talking about her use of illegal drugs to pain-manage; the patient picks the cards up and holds them against her body, taking ownership of them. She has brought in ten cards and is almost shuffling them—is she handling them for security or taking ownership at this point? She holds the cards while indicating pain in her stomach for quite a long time without actually revealing the images. A little later, at about 39 min, the doctor invites the patient to talk about the cards she has chosen*’. The pain specialist describes the doctor in the extract as a very ‘*competent pain physician, articulate, reassuring, allowing the patient plenty of time to talk*’, giving as example the way the ‘*doctor invites the patient to use the cards at the beginning or any time during*’, explaining how ‘*the patient plays with them but only when prompted at 39 min into the consultation does she show the cards and explain her choice*’. (The pain specialist adds that ‘*the patient is also articulate and has seen a very wide range of healthcare practitioners, orthodox and complementary*’.) Almost in contrast to the ‘*quietly absorbed*’ interaction and ‘*reassuring*’ doctor, the art therapist emphasises the tensions in a ‘*difficult exchange in which the patient is upset and angry*’. Although the pain specialist has described the doctor as ‘*non-judgmental, checks understanding, uses metaphors to explain chronic pain and is very patient and interested*’, the art psychotherapist describes how ‘*the doctor has to ask if the patient will speak about the cards. The patient is at first quite controlling of the cards, their position, tone and pace, which lets her manage how much she will share of herself*.’

These multiple interpretations raise several questions: who has initiated use of the cards, who has agency at this point, what shifts are the images facilitating, how can the emotional landscape be characterised, how does that impact on the exchange, and what do both participants take away from the encounter?

## Transactional objects

The psychologist, the art psychotherapist and the artist commented on the effect of images as tangible objects in the room. The psychologist observes that ‘*at the point where the transcript starts, an observer who had just arrived would see that the patient holds the stage, while the clinician listens actively, concerned. In fact, the doctor has moved closer than at the start, while the patient has moved little except to become more upright in her chair, a confident stance. After a momentary hesitation, the patient puts the fourth card down decisively in front of the doctor, although her hand stays for a moment over the card as if she might take it back. Then she states, with some hesitation, that it is to do with her self-identity. While she says this, she holds the remaining cards in both hands in front of her, in a somewhat closed posture, but looking at the doctor. The doctor holds the image in both hands, directly between herself and the patient. When she asks about what about the image makes the patient refer to self-identity, the doctor leans forward further and puts the card on the desk so both can see it the right way up*’. The art psychotherapist interprets this with reference to ‘*Schaverien’s theory of transactional objects*,^11^ *in which images are handled and used concretely by the client to control aspects of the therapeutic relationship: here, how much the patient will choose to reveal of herself to the doctor*’. The patient holds onto the cards throughout most of the card use, controlling the pace of their interaction—handing them one by one to the doctor who looks at them and lays them on the desk between them. The artist observes that '*the materiality of the images could be seen as bringing patients’ experience directly into the consultation. Images are affecting and visceral in their directness and open up a different kind of space from the traditional verbal encounters within medical pain consultations. There is a lot of physical handling and touching of the face and of the images, also noticeable in other consultations, where patients make claw-like gestures, hold the side of their face, pinch their body, etc, with the effect of bringing pain as a physical sensation into the consulting room. Both speakers become involved in looking back and forth between each other and the image forming a clinician-patient-image triangle’*.^11^ The art psychotherapist observes here ‘*that the exchange shows the two participants adjusting to each other, leading to what seems to be a meaningful and useful communication about the emotional and physical aspects of the patient’s experience of pain*.’

## Identity building and figurative language

Rather than describing physical sensation such as temperature, which she could easily have used the card for, the patient chose to use the image to describe how pain has erased and changed her identity. In the previous section of the consultation, she offered many identity-building statements, for example, in relation to being a creative professional, the calibre of the people she works with, a person who eats seeds on her food, which seem to lead up to this extract where she highlights change in identity as a major cause of distress, reinforced later in the consultation by comments about how her opioid use makes her feel ‘*dirty*’, like a *‘junkie’* and ‘*not sharp’*. When prompted for more detail by the doctor, the patient explains that, in her interpretation, the fact that the person’s face on the card looks as if it is ‘burning off’ suggests something about self-identity. In other words, she interprets the image as a metaphor for a particular aspect of her experience of chronic pain.

The linguist observes that ‘*the face is conventionally associated with one’s individuality: it is the body part that tends to be almost always exposed and that is most immediately recognisable. The fact that part of the person’s face in the card is burning is therefore interpreted metaphorically as a negative change in the patient’s own perception of herself, due to her pain. The patient relates this interpretation to her own personal characteristics and professional activities (“I’m a visual, I work in visual”). At the end of the extract, the burning in the picture is also made relevant to the perception of temperature associated with the patient’s pain (“plus my pain is hot”).’*

**Figure 3:**
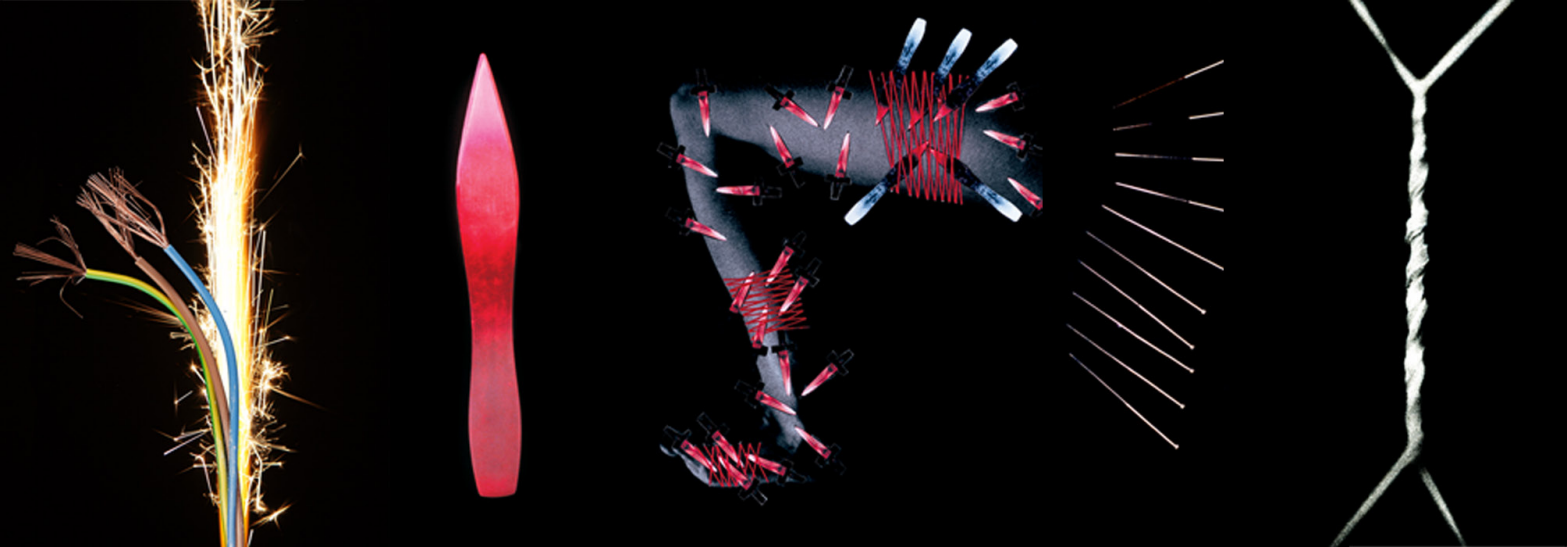
Cards selected by this patient to take into her consultation. All images co-created by the Deborah Padfield with patients with chronic pain.

**Figure 4:**
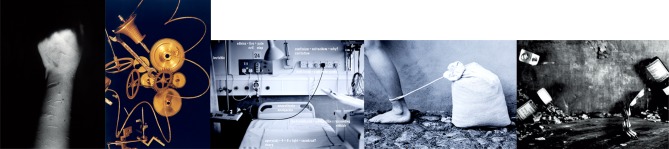
Remaining cards selected by this patient to take into her consultation. All images co-created by Deborah Padfield with patients with chronic pain.

In order to investigate the generalisability of these observations, we used the semantic annotation tool in the online software package Wmatrix^12^ to compare all interactions around the cards in the 17 consultations with the rest of those interactions where the cards are not used. Through this the linguist found that ‘*the types of words that were found to be used much more frequently around the cards include words related to temperature (eg, ‘burning’, ‘fire’) and to electricity (eg, ‘electric shock’). In context, these consistently turned out to be figurative descriptions of the quality of the patients’ pain. These were first introduced by patients and then often repeated by clinicians. In addition, we found that the word ‘like’ is used much more often around the cards than in the rest of the consultations. This involves particularly the use of ‘like’ as a preposition to introduce a simile, as in “like a knife going through me” and “this is when I’m completely like a rag doll”. Overall, therefore, there is evidence that the language used around the cards is characterised by figurative descriptions of pain, and of the impact of the pain on the person’s life more generally. In other words, what we observed in the extract above is in fact common in our data more generally’*. Building on this, the artist noticed how dramatic the language around the cards was throughout this encounter, ‘*suggesting active processes such as fire burning, wires and sparks flying, medication flying, elastic bands twisting, a leg leaning away from a burden, mechanical pieces unravelling, knives going into the leg, words over a hospital bed not making sense, resisting meaning and a consistency in the colours within the images selected, for example, yellow, orange, red and black—the colours of fire*.’ (see figures 3 and 4 showing all cards selected). Physician and literary scholar David Biro^13^ argues that because of a lack of language for pain, people resort to metaphor, to describing something intangible, invisible and difficult to articulate through something more familiar and concrete. Many of the components of pain metaphors Biro identified are present within the visual metaphors our patient chose, and specifically within fire, for example, agent, weapon, force, capacity, injury and damage—part of an active injury inducing process with conjacent symbolic meaning.

The art psychotherapist’s perspective enhances our understanding of the impact of visual metaphors in a relational context, focusing on the dynamic evolving nature of a consultation: '*Broadly, in psychodynamic terms, we could say the patient is projecting something about their experience onto the image, imbuing it with their own meaning, so that it comes to symbolise some aspect of herself or her experience. This can be seen as a process of introjection and projection, as we take in aspects of the image, which exist in the outer world, and bestow aspects of our inner world on it, the two are in constant dynamic interplay*.

*Art psychotherapist Skaife*^14^ *has argued that an image’s meaning is made intersubjectively in the context of the moment rather than being a static representation of an inner world, and she asks what the image brings in to that particular situation. Seen like this, the image’s meaning is context-dependent. Fire could be warming, destructive, creative or cleansing, but here (as the linguist points out) the effect is that the face is in flames. People on fire might call to mind protest (setting the self on fire) or persecution (burnt at the stake), for example. In this context, the patient’s words about the image accentuate the act—something terrible is happening to this woman: ‘that person’s face is burning off.’ In addition to ‘self-identity’, the image brings something of the patient’s anger and distress into the room. The disturbance is heightened by the burning woman’s expression, which is outwardly calm through apparent suffering (burning), resulting in a visible disconnect between inner and outer, and between the two sides of the face. We could see the image as reflecting the patient’s experience of pain as a violent attack on her identity that causes her extreme emotional suffering. Her emotional well-being is a key theme that the doctor later picks up on, also reflected in the treatment plan where the patient has access to psychological therapies*.’

In contrast, the pain specialist feels that the patient’s own story, although beginning to emerge via the cards, may have been better elicited had the cards been used earlier. She wrote, ‘*The doctor was very interested in the use of the cards and their interpretation but, reviewing the consultation, I wonder what would have emerged if the cards had been engaged with earlier. Although significant facts in the patient’s history had been elicited prior to the use of the cards, the cards seem to offer another opportunity to review these in a more psychosocial approach. However, they were not used till the end of a long consultation and with time being a major consideration there was less time than she might have liked to develop this emerging theme.’*

This draws attention to the challenge of inviting emotional disclosure within a limited time frame with the conflicting needs to explore and to close it down sufficiently before the consultation ends. Arguably, used earlier in the consultation, the cards could speed up disclosure of relevant information and of the social and emotional impact of pain.

## Emotional disclosure

Metaphors are frequently used to communicate subjective and poorly delineated experiences, particularly emotional states and processes. In a narrative analysis of the five most frequently used narrative themes, emotional disclosure predominated. In all our sets of data, patients’ narratives around the cards exposed the emotional components of pain, drawing on themes of loss, anxiety, fear, shame, disintegration and even suicidal feelings. In some consultations, these were revisited later by clinicians. In our extract, the pain specialist observed that ‘*until the cards are used there was only one significant instance when the patient became emotional. This was about the death of her mother, with which the doctor deals very well. The doctor shows great interest in the cards and makes little attempt to provide her own interpretation, she uses the patient’s*.’ She also observes that the cards brought out the ‘*impact of the pain and potential for self-harm. Although the doctor tries to establish whether this was a serious remark, the patient brushes it off by saying it was a flippant remark. I had the feeling that there was more to this remark and choice of card. She also chooses a card with multiple knives, and describes waking at night and wanting to put a knife into her abdomen to get a different type of pain, but possibly also that it would make the pain more visible, attract attention?’* This parallels the artist’s earlier observation about the dramatic nature of the language the patient uses and the active processes of destruction she references.

The linguist noticed the way in which the ‘*patient interprets the card metaphorically to reveal to the doctor something about the consequences of her pain for her own mental and emotional life, that is, the perception of a negative change in her own self-identity. This use of the cards to disclose emotionally charged and sensitive experiences occurs with other images selected by the same patient, and also more generally with other patients. The computer-aided comparison between all interactions involving the cards and the rest of all the consultations combined revealed that, when discussing the cards, patients use the words “feel” and “feeling” much more frequently. More specifically, they use these words both to introduce an aspect of the physical sensation of pain (eg, “I feel a burning in my mouth”), and to reveal emotional and deeply personal experiences (eg, "I feel lost" and “I feel as if everything is coming apart”). More generally, when explaining the significance of the cards, some patients reveal details of their own lives that they are ashamed of (eg, repeatedly lying to friends to avoid going out) and occasionally allude to potentially suicidal thoughts (eg, “it’s my I’ve-had-enough days”)*’.

Building on this, the artist notes how as ‘*material objects the photographs become embodiments of pain with physical resonance within the consulting space, they create connections between dialogue participants and between the emotional and the sensory; the mind and body. This reflects the linguist’s observations of the use of the word ‘feel’ to describe both ‘bodily sensation and emotional experience*’.

## Agency

The linguistic analysis reveals one of the most tangible impacts of the images on patient-clinician interaction—that when the cards are used, patients speak more. It illuminates how ‘*in this extract, the patient speaks approximately 50 per cent more words than the doctor (72 words vs 46, where words are defined orthographically). If we consider the whole of the interaction about the cards in this particular consultation, however, the relative difference in the respective volume of talk is even bigger: the patient speaks almost four times more words than the doctor. In contrast, when the cards are not being used, the patient and the doctor speak roughly equal numbers of words*.

*Again, what applies to this patient was found to apply more generally in our data. Taken all together, the 17 patients in our complete data set speak 8463 words around the cards, while the clinicians speak 5188, that is, the patients speak just over 60 per cent more words than the clinicians when the cards are actively being used. In contrast, when the cards are not being used, the clinicians cumulatively speak 10 per cent more words than the patients, that is, 62 024 words vs 55 617. In other words, the proportion of patient talk versus clinician talk consistently increases when the cards are being used*.’

The artist argues that what this suggests is twofold. '*First, there is the possibility that the cards themselves have agency. They have been co-created with other pain patients and so could be seen as placing the bodies of other patients within the communication process. In another consultation for example, after using the cards, one patient says “At least I know I am not on my own”. In anthropology, there is a general move from Gell, through Tilley, Miller and Pinney to consider and give importance to the materiality of art objects. Gell’s notion of the art object as relational*^15^ *provides a key insight into the way images work in social spaces such as the consulting room or the workshop. Handling, viewing and responding to the pain cards could be viewed as performances of identity construction and relationship building, which appear to be enacted during this extract and the card use during this consultation as a whole. On the other hand, the images could be seen as conferring agency on the patient who, in this extract, takes control, laying each card down on the desk one by one. Another way of understanding what is happening via the cards would be to see them as transitional objects in the Winnicottian sense*’. The art psychotherapist helps us see how ‘*Winnicott’s ideas about transitional objects and spaces*^16^ *can help us think about the image as occupying an “in-between” place. They are a way of describing an in-between area where our subjective inner world experience meets the external world. The images are like transitional objects in that patients are projecting their own subjective inner experiences onto something that exists concretely and externally (the image). We can think that the images are imbued with projected meaning and therefore contain the stuff of the patient’s inner world as well as existing materially, as perceived by both participants in common, to be looked at and thought about*’. Does this reveal one of the paradoxes of pain: that it is a crisis in meaning-making and that at no other point is the gulf in significance of a pain wider than in pain encounters, and at no point is it more important to cross?

## Non-verbal interaction

The psychologist, exploring the non-verbal component of the interactions, observes how by the point where ‘*the patient mentions the face “burning off” her voice becomes quieter and softer, and she maintains that tone until the end of the excerpt. On stating that it is “a portrait of a person”, she and the doctor hold one another’s gaze and there is a moment of complete stillness, after which the patient becomes more animated, gesticulating as she describes herself as a visual person; she starts another explanation about the face “burning off” but does not complete it, and ends with a concrete association of the pain feeling hot’*.

The art psychotherapist helps us make sense of the non-verbal analysis when she observes how it ‘*brings our attention to the clinician’s placing of the image the right way up on the desk between them, so that both participants can see it. Looking at the image between them and at each other requires joint attention skills. We can draw on Isserow’s ideas*,^17 18^ *which suggest that joint attention to the image is a key feature of art therapy, which creates a “triangular” relating pattern as both people are involved in looking back and forth between each other and the image, to try to understand the feeling of it and gauge its meaning. Isserow considers psychoanalytic theories of triadic (as opposed to basic dyadic) relating, which require a more sophisticated relating to the world and others needed for symbol formation, and he links achieving this with developmental theories of mind, which involve the ability to acknowledge that another person also has a thinking mind with another point of view. His work suggests using images, which encourages joined up triadic relating and joint attention skills, can lay the grounds for reflective self-awareness, symbolisation and communication’*.

*We see here what seems to be ‘reflective self-awareness’*.^17^ *The psychologist relates how the ’patient’s voice becomes softer as she describes ‘a portrait of a person’. We can see that a meaningful emotional connection appears to have been made, and the patient seems to reference this human connection by drawing attention to the ‘humanness’ of the image. It is ‘a portrait of a person’ and perhaps unconsciously to herself as ‘a person’, with parallels between the picture as portrait, and the portrait of herself she now communicates to the clinician’*.

In our non-verbal analysis, we looked for affiliative behaviours drawn from the literature, for example, smiling, meeting the other’s gaze, reducing distance between the two (eg, by leaning forward), nodding, speaking gently, rather than the opposite behaviours of frowning, turning away, showing impatience or other disengagement—and dominance behaviours—postural and gestural expansion, looking at the other only when speaking, speaking loudly and interrupting and asymmetry in posture rather than the opposite behaviours of avoiding gaze, postural constriction and speaking quietly.^19^ The psychologist observed how ‘*the axes of affiliative and dominance behaviours are orthogonal, and the norm is for the clinician to show dominance that elicits submissive behaviour from the patient, while affiliation is matched and often increases as clinician and patient get to know one another. Affiliative behaviours are very evident in this excerpt, particularly in mutual gaze and unconsciously mirrored postures, while both patient and doctor momentarily show a few small dominance behaviours but without eliciting deference from the other. As in our analysis of all consultations with images*,^2^ *clinicians responded with affiliative behaviours to patients’ presenting and explaining their chosen images, and those affiliative behaviours corresponded better when images were present than when they were not. Although in this excerpt the engagement shown by the doctor was already at a consistently high level, these patterns are visible in the short excerpt*.’

Medical discourses are likely to skew the power relations, with the clinician in the powerful ‘expert’ position. In this complex example, the art psychotherapist observes that the patient seems to have ‘*sometimes used the images to control their interaction, and the tensions of their former exchange are present, although she makes some significant disclosures and we could think some trust is developing. However, as participants consider* figure 1 *together something different happens; as they study the image on the desk between them, the doctor comes out of her role and appears to give increased authority to the patient, saying that the patient has helped her to think differently about another patient of hers who originally made the image: “… it helps me identify with that as well”. This is surprisingly exposing of the doctor, her thought processes and previous work with other patients, “I think it was her… um”. It seems to have a levelling effect and empower the patient, who then asserts her own expertise “I work in visuals”, drawing on her own strengths and knowledge, and taking a new identity as an expert. We can use art psychotherapist Tipple’s research here*,^20^ *which proposes the image as something around which role and hierarchy can be negotiated. Drawing on art historical ideas of exchange or barter, Tipple*^20^ *suggests that using images allows participants to negotiate and propose identity between them. These identities shift as the subjectivities of both participants are formed and reformed from moment to moment during the interaction*.’

**Figure 5:**
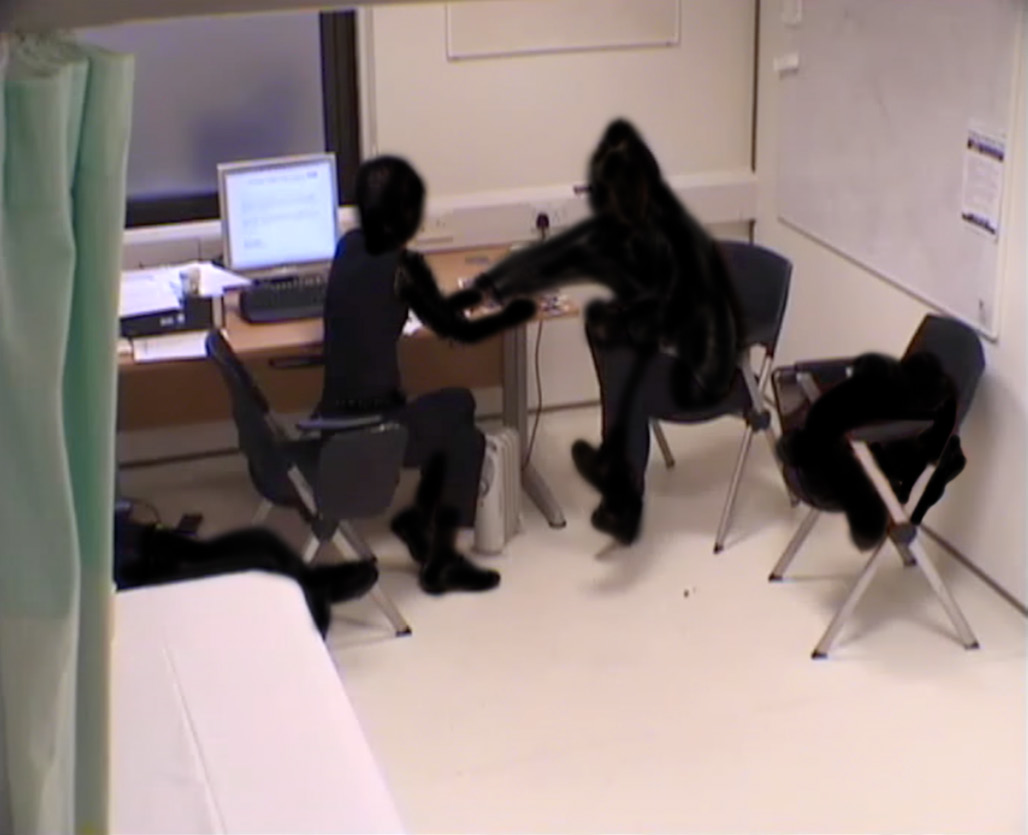
Anonymised screen grab of video footage from this consultation (filmed with consent) showing the non-verbal interaction.

In contrast to the negotiated interaction suggested by the art psychotherapist, the pain specialist felt that there was ‘*perhaps apprehension about the use of the cards as neither clinicians nor patients had used them before and many clinicians had not had time to look through the whole pack. It was a totally new experience. Just as patients have been able to draw the clinician to a computer to make a point so these images are potentially enabling a patient to say something they could not or did not know how to say in words alone. Some clinicians actively include the computer in the consultation (triadic) whereas others largely exclude them (dyadic)*.^21^ *These same effects may have been seen with the use of the pain cards*.’ The artist observes that ‘*the non-verbal behavior moves from participants being on either third of the frame at the beginning of the consultation, through the clinician leaning towards the cards and the patient (end of our extract) to, at the end of the consultation, both patient and clinician inhabiting the middle space, pouring over the cards together—moving them around on the desk—touching them and activating the central space between them*’(figure 5).

Of the feedback offered by the doctor in relation to information elicited by the cards, the linguist notes that she ‘*provides back-challenging feedback several times (“ah okay”, “right okay”), asks for clarification about the patient’s comment about self-identity, provides an explicit positive evaluation (“that is interesting”) and mentions why she is in a particularly good position to relate to the patient’s point about self-identity (turn 8). In turn 12, she also repeats the patient’s words (“visual”). This tendency to provide positive and empathetic responses was observed more generally in interactions around the cards across the 17 consultations. The computer-aided comparison between the interactions around the images and the rest of the consultations shows that clinicians use words suggesting positive evaluation much more frequently around the cards (eg, “fascinating”). Like the patients, they also show a statistically significantly higher usage of words such as ‘feel’, but in most cases the verb is used in reference to an experience of the patient’s, whether to ask for clarification or to show understanding (eg, “This is about how you feel frustrated and tense, yes?”). Moreover, as we have already mentioned, consultants also show higher frequencies of metaphorical descriptions of the quality of the patient’s pain, again to check or confirm their understanding of the patient’s experience (“Okay. So it’s burning, sharp, yeah?”)*.’ This further exemplifies the images as a third space, a shared reference point within and against which to check meaning.

## Conclusion

*‘In recent years, the health-arts nexus has received increasing attention from clinicians, researchers, healthcare/social care professionals and policy-makers. However, evidence of the relationship between arts engagement and population health is in its infancy*’.^22^ This paper argues that bringing together multiple perspectives and methodologies improves understanding of the impact of the arts and their practices on medicine (and vice versa), and provides much-needed evidence of the benefits of such exchange. Multiple meanings arise from the diverse sources. The linguistic analyses highlight changes in language when the cards are used, such as an increase in metaphoric and emotional language and the proportionate number of words spoken by the patient; the non-verbal analysis clarifies the increase in affiliative behaviours when the cards are used. The art-psychotherapeutic theory helps us understand how and why these changes happen, drawing attention to the triangle created by the introduction of an image into the encounter, the negotiation between inner and outer worlds it facilitates and the evolving dynamic nature of the consultation. Layered on these, the insights of fine art and medical practitioners focus on material and relational qualities of the image, the multiple interpretations possible in response to both an image and/or a metaphor, the resultant triadic and negotiated nature of the exchange and ways in which the images reinforce a psychosocial approach to medical dialogue. The result is a rich account of a short extract from a complex encounter, identifying paradoxes present not just within the experience of pain but the exchanges which happen around it. Integrating knowledge from such diverse disciplines allows us to highlight the tensions and paradoxes within pain encounters while improving our understanding of them. The authors argue that exploring meaning is an essential part of understanding pain better, and that images introduced into an encounter become catalysts for both meaning-making and change. The combination of detailed analyses and broader findings the paper provides builds a complex picture of the multifaceted, contradictory and paradoxical nature of pain experience, the drive to communicate it, and what is at stake for the sufferer. It acts as a first step towards acknowledging and learning from differences in meaning, with implications for future cross-cultural as well as multidisciplinary work.

‘*The way we respond to people-in-pain strikes at the heart of what it means to be human*.^23^
